# Retrieval-Augmented Language Models for Clinical Decision Support in the Classification of Inborn Errors of Immunity

**DOI:** 10.1007/s10875-026-02035-9

**Published:** 2026-06-05

**Authors:** Afrooz Arzehgar, Saeed Varasteh Yazdi, Hamid Ahanchian, Saeid Eslami

**Affiliations:** 1 Department of Medical Informatics, Faculty of Medicine, Mashhad University of Medical Sciences, Mashhad, Iran; 2 Department of Operations, Data and Artificial Intelligence, emlyon business school, Lyon, France; 3 Allergy Research Center, School of Medicine, Mashhad University of Medical Sciences, Mashhad, Iran; 4 Department of Medical Informatics, UMC-Location AMC, University of Amsterdam, Amsterdam, Netherlands

**Keywords:** Inborn errors of immunity, Primary immunodeficiency, Large language models, Retrieval-augmented generation, Clinical decision support

## Abstract

Early diagnosis of inborn errors of immunity (IEIs) can make a difference in patient outcomes and even cut healthcare costs. However, there are some challenges to overcome, such as clinical complexity, low awareness, and limited resources. Generative artificial intelligence has attracted considerable global attention in medical domains, particularly when integrated into clinical decision support systems (CDSS), as it has the potential to facilitate data interpretation, clinical reasoning, and the optimal use of knowledge resources. Preliminary studies have explored the potential of large language models (LLMs) in various information retrieval tasks, but a systematic evaluation of LLMs with and without retrieval mechanisms for IEI classification is still unexplored. We evaluated and compared the validity and reliability of the responses generated by four open-source and closed-source LLMs, in their baseline form and with augmented data, across 169 IEI patient records, using two input scenarios and four prompt templates. Our primary finding was that the models varied in terms of reliability and performance. The most reliable models were Gemini-1.5-Pro and Llama-3.1-8B-Instruct (*κ =0.98*) and the best-performing model without data augmentation was Gemini with an F1 score of $43.39\, \% \pm 0.10$. The results also showed that retrieval strategies improved the average classification performance, increasing the F1 score from $34\, \%$ to $53\, \%$ across all models. DeepSeek-R1, which reasoned over retrieved information through the integration of quality refinement and structured retrieval, achieved the best weighted F1 score of $66.94\, \% \pm 1.19$. The study highlights the effective use of generative AI and retrieval-augmented models as a decision support tool for IEI classification. However, incorporating retrieval systems into clinical decision-making processes requires adequate input, effective prompt engineering, and the adoption of retrieval strategies.

**Supplementary Information:**

The online version contains supplementary material available at 10.1007/s10875-026-02035-9.

## Introduction

Inborn errors of immunity (IEIs), represent a diverse group of over 500 rare genetic disorders resulted from monogenic germline defects that impair immune function and predispose individuals to recurrent infections, malignancies, and autoimmune or autoinflammatory conditions [1–3]. Although individually rare, the burden on patients and healthcare providers poses substantial public health problems [1, 2]. Without early diagnosis and treatment, IEIs result in increased costs of healthcare and a wide variety of serious complications, including life-threatening infections, developmental defects, organ-specific diseases, malignancies, and increased mortality [4].

Early and accurate diagnosis of IEIs presents many challenges due to considerable genetic heterogeneity and highly variable phenotypic expressions across the disease spectrum [3, 5–7]. It often requires analysis and integration of clinical data sources, such as family history, vaccination and treatment reactions, surgical and medical procedures performed, sensory or motor functions, immunophenotyping, and other clinical and paraclinical information [8]. The low prevalence, general lack of awareness, and limited resources and clinical experience, especially in areas with few immunologists, can also increase diagnostic complexity and lead to inaccurate diagnostic results, adverse patient outcomes, and increased burden of IEI disease [9, 10].

Artificial intelligence (AI) approaches demonstrate significant potential to address IEI challenges. ML-based risk scoring and IEI classification can be based on structured data from electronic health records (EHRs) or registries [11]. Some studies have shown that machine learning (ML) techniques are more effective than traditional statistical methods [12, 13]. Furthermore, gene-specific models were developed and integrated into clinical workflows for underrepresented populations, leading to improved classification and the potential reduction of healthcare disparities [14]. For allergists and immunologists, AI offers enhanced diagnostic precision and enables more personalized treatments [15], and can also be incorporated into clinical guidelines. One example is the immune deficiency foundation’s warning signs and expert-guided scoring system [8].

Natural language processing (NLP) [16–18] and, more recently, generative AI [19] have become valuable tools for IEI diagnosis, management, and research. With their capabilities for converting unstructured clinical text into structured data and performing powerful reasoning, they can support clinical decision-making in complex and rare conditions, including IEIs. These models through their embedded medical knowledge, advanced NLP techniques, and reasoning process, holds potential to address the diagnostic needs and challenges [20–22]. Moreover, retrieval-augmented generation (RAG) frameworks, including standard RAG [23–27], corrective post-retrieval approaches [28], and pipeline-based methods with document re-ranking and refinement [29], have demonstrated enhanced factual accuracy and contextual relevance in complex clinical settings. RAG strategies provide relevant evidence in response to queries. The process integrates specialized and up-to-date knowledge as a supportive context into reasoning processes of LLMs, enhancing the pretrained baseline LLM capabilities.

Although the general capabilities of LLMs and RAG frameworks in medical contexts are being evaluated independently [30–34], their efficacy in rare and genetically diverse conditions, such as IEI, requires further investigation. Given the rare and complex nature of IEIs, we hypothesize that carefully designed RAG framework can assist in the IEI diagnostic classification. To our knowledge, there has been no systematic evaluation of baseline LLMs and RAG strategies for the IEI classification. This study aims to develop a classification pipeline and evaluate baseline LLMs and RAG strategies for IEI class-level categorization. The primary objective is to evaluate the classification performance of open-source and closed-source LLMs by examining them when augmented with query-related evidence. The secondary objective is to compare the ability of baseline LLMs and RAG strategies to generate consistent responses to user queries. The tertiary objective is to compare the performance of baseline LLMs and RAG strategies with medical students. This systematic evaluation provides insights into how pretrained baseline LLMs generate diagnostic classifications using internal knowledge alone, and how retrieval augmentation affects the validity and reliability of those classifications by enhancing the model’s ability to integrate external, context-specific information.

## Materials and Methods

An evaluation framework was designed using four prompt strategies, applied both with and without retrieval augmentation, to assess the ability of LLMs to categorize patient cases into IEI disease classes. The study involved querying several state-of-the-art LLMs with structured information from native patient medical records. Five RAG strategies were implemented and compared, in which relevant, query-related evidence was retrieved, ranked, processed, and augmented to the input prompt. Moreover, a comparison with medical students’ evaluations (interns and residents) was conducted.

### Study Subjects

A dataset of 169 medical records, originally documented in Persian, from native patients diagnosed with IEI was collected at Akbar Hospital in Mashhad, Iran, between 2022 and 2024. The dataset includes demographic and clinical information, such as sex; parental consanguinity (including the specific degree of relation); and age at both the time of data collection and onset of first presentation (all recorded in years, months, and days). The mean age of first presentation onset was *1.64 ± 2.56* years. The IEI classes for each patient were confirmed through genetic testing. The descriptive statistics of the dataset are presented in Table 1.

**Table 1:** Characteristics of 169 patients diagnosed with IEI in the dataset

Variable		Category	Count (%)
Gender		Male	98 (58.0)
		Female	71 (42.0)
Consanguinity		Yes	97 (57.4)
		No	72 (42.6)
Family history		Yes	149 (88.2)
		No	20 (11.8)
Most reported symptoms		Pneumonia	74 (43.8)
		Otitis	20 (11.8)
		Severe fever	18 (10.7)
		Lymphadenopathy	14 (8.3)
		Eczema	13 (7.7)
		Disseminated BCG	13 (7.7)
		Diarrhea	8 (4.7)
Diagnosed IEI classes	C1	Combined immunodeficiencies with associated or syndromic features	44 (26.0)
	C2	Congenital defects of phagocyte number or function	46 (27.2)
	C3	Predominantly antibody deficiencies	34 (20.1)
	C4	Immunodeficiencies affecting cellular and humoral immunity	30 (17.7)
	C5	Defects in intrinsic and innate immunity	9 (5.3)
	C6	Diseases of immune dysregulation	4 (2.3)
	C7	Autoinflammatory disorders	1 (0.6)
	C8	Phenocopies of inborn errors of immunity	1 (0.6)
	C9	Complement deficiencies	0 (0.0)
	C10	Bone Marrow Failure	0 (0.0)

The clinical data included detailed documentation of each patient’s first presenting symptoms and a family history, covering reports of diseases or deaths among relatives potentially associated with immunodeficiency disorders. Laboratory results included complete blood count parameters, such as white blood cell (WBC) count, lymphocyte count and percentage, polymorphonuclear neutrophil (PMN) count and percentage, eosinophil count and percentage, hemoglobin concentration (g/dL), platelet count, and serum immunoglobulin levels (IgM, IgG, IgA, and IgE), with all values standardized to appropriate units. Immunophenotyping data included the relative percentages of key lymphocyte subsets, including CD3+ T cells, CD4+ helper T cells, CD8+ cytotoxic T cells, CD19+ B cells, natural killer (NK) cells (CD16+CD56+), as well as cells expressing CD11a+ (integrin *α *L) and CD18+ (integrin *β *2). The dataset also included results of the nitroblue tetrazolium (NBT) test, used to assess neutrophil oxidative burst activity.

### Data Preprocessing

As the original records were in Persian, a systematic translation and structuring process was performed. Clinical experts reviewed the raw records, extract key diagnostic features, which were then standardized into English using consistent medical terminology according to SNOMED CT standard. This ensured that the input forms provided to the LLMs utilized a harmonized vocabulary for symptoms and laboratory parameters, thereby reducing linguistic ambiguity during the reasoning process.

**Fig. 1:**
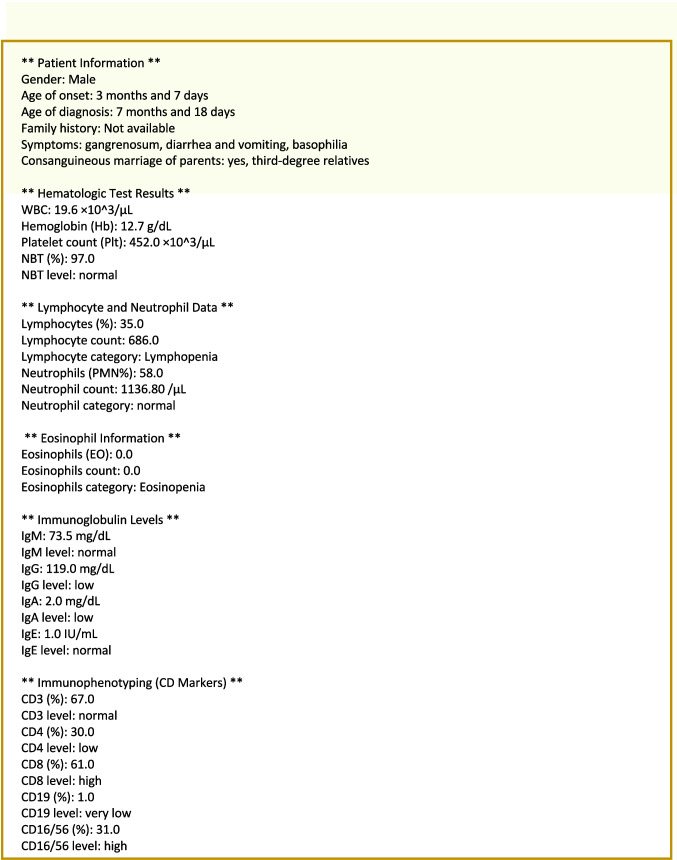
Sample patient information. The highlighted parts are used in the minimal form, while the entire information is used in the full-form input

IEI classes were mapped to the standard 10-category classification. In this cohort, certain categories are absent or nearly absent: C9 (complement deficiencies) and C10 (bone marrow failure) are not represented (*n=0*), and C7 (autoinflammatory disorders) and C8 (phenocopies of IEI) are represented by single cases (*n=1*). These classes were retained in the classification label space to simulate the full complexity of IEI class assignment and to evaluate model specificity. All patient information was structured into demographic, clinical, and laboratory information. For each patient, two input forms were generated (see Fig. 1 for an example):

- Minimal: This form included only basic clinical information, such as age, sex, family history, consanguinity of parents, and first presenting symptoms. It simulates a minimal input scenario where only initial patient information is available, excluding any laboratory data.
- Full: This extended format included all elements from the minimal form, plus the detailed laboratory results. It included laboratory findings from hematology tests, as well as counts of lymphocytes, neutrophils, eosinophils, immunoglobulins, and cluster of differentiation (CD) markers. This format provides a specialist-level clinical evaluation with a more comprehensive clinical context.

The data preprocessing pipeline involved anonymization procedures to protect patient confidentiality and standardization and harmonization of medical terminology and units of measurement across all records. The information regarding the laboratory values and ranges are reported in Appendix D.

### Baseline LLMs

Four LLMs were selected for comparative evaluation: Llama-3.1-8B-Instruct (**Llama**) [35], DeepSeek-R1 (**Deepseek**) [36], GPT-4o-Mini (**GPT-4o-Mini**) [37], and Gemini-1.5-Pro (**Gemini**) [38]. All closed-source models were accessed between June and July 2025. The selection of these models was made to provide a representation of both open-source and closed-source, with a range of model complexities, architectural designs, and pre-training datasets. All models were utilized in their publicly released, general-purpose instruction-tuned versions, without any additional domain-specific fine-tuning or adaptation. This approach ensured a standardized assessment of each model’s zero-shot diagnostic reasoning capabilities. To minimize variability and promote deterministic outputs, the temperature was set to 0.1 for all models. Top-p and maximum output tokens were left at their respective model defaults. A predefined set of prompts was used to query each model with structured clinical case information. Each model was instructed to output a predicted IEI class label (C1–C10) and an associated confidence score for its prediction.

**Fig. 2:**
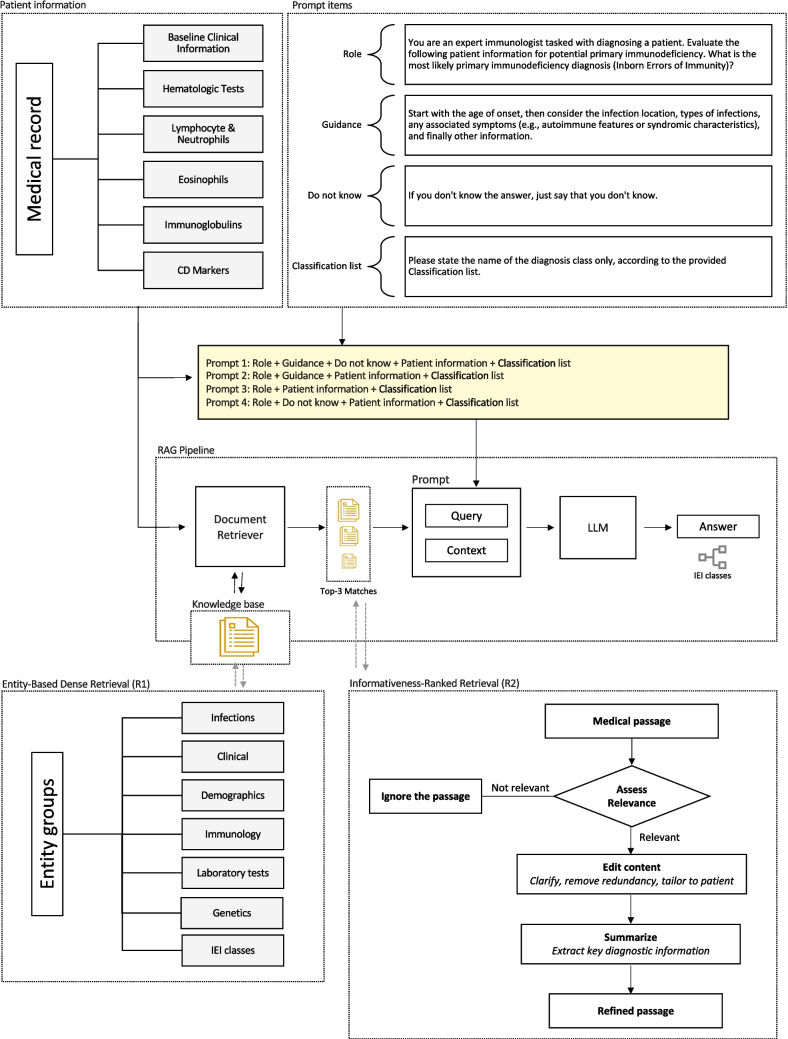
Block diagram of the proposed RAG strategies

To explore the effect of prompt strategy on model performance, four prompt strategies were designed to act as an expert immunologist for diagnosing IEI classes. The prompts included two primary types: (1) direct classification, and (2) classification with step-by-step diagnostic reasoning. Each format had two variations: one requiring a response from predefined IEI classes, and one allowing the model to respond “I don’t know” when the available information or internal knowledge was insufficient for class assignment. The four prompts are listed in Fig. 2.

### Retrieval Augmented Generation

To evaluate the impact of knowledge source integration on diagnostic classification performance, several RAG pipelines were implemented. Pipelines consisted of three key components: (1) indexing a domain-specific knowledge base, (2) retrieving relevant evidence based on patient information, and (3) processing and augmenting the retrieved evidence into the LLM input to support diagnostic reasoning. The knowledge base used for retrieval consisted of two primary sources: the 2022 International Union of Immunological Societies (IUIS) expert committee update on the classification of IEI [2] and Stiehm’s Immune Deficiencies reference textbook [39].

The knowledge sources were processed into two formats: raw text chunks of 1,000 characters, and structured entity-based representations of clinically relevant concepts. Entity extraction was conducted using predefined categories, including infections, clinical features, demographics, immunology, laboratory tests and diagnostic procedures, genetic data, and IEI classes. A task-specific prompt guided an LLM to identify and extract clinically relevant concepts from both the raw knowledge source chunks and the patient records. By mapping both patient queries and the knowledge base into this shared structured form, the retrieval strategy performed matching based on clinical relevance rather than simple lexical overlap. The complete raw JSON structure and hierarchy of these entity groups are detailed in Appendix E. Five retrieval strategies were evaluated within the RAG pipeline:

- **Sparse Retrieval**: This method used the classic Best Match 25 (BM25) [40] ranking function, a well-known sparse retrieval algorithm based on term frequency–inverse document frequency (TF–IDF). It ranked knowledge base chunks according to their lexical overlap with the patient information query.
- **Dense Retrieval**: This method used patient information and knowledge base chunks, which were encoded into high-dimensional embeddings using a pre-trained embedding model [41]. The top-*k* relevant chunks were further extracted and sorted by using cosine similarity in the embedding space.
- **Entity-Based Dense Retrieval (R1)**: This method modified the dense retrieval strategy using the entity-based structured form. Entities of patient information were extracted, which were then matched against pre-extracted entities of the chunks of the knowledge bases [42, 43].
- **Informativeness-Ranked Retrieval (R2)**: This method applied a filter to exclude irrelevant chunks. Each retrieved chunk from the knowledge base was assigned an informativeness label and pre-processed using the LLM to generate a concise and refined summary. Non-informative or redundant content is filtered out, and only the most clinically relevant and refined summaries were considered. This strategy prioritized concise content that contributes directly to diagnostic reasoning [44, 45].
- **R1+R2**: This hybrid method integrated the semantic precision of the R1 strategy with the content quality refinement of the R2 strategy. First, relevant entities from patient information were matched with structured entities in the chunks of knowledge base. Then, the top-*k* chunks were passed through the informativeness-ranking filter to retain only the most meaningful and contextually rich content. This hybrid strategy was designed to maximize both relevance and informativeness.

Baseline LLM and RAG pipelines were implemented in this study using Python and the LangChain framework. For dense retrieval, OpenAI’s Text-Embedding-3-Small model was employed. In all experiments, the retrieval step was configured to return the top three (*k=3*) most relevant chunks. Figure 2 illustrates the architecture of the proposed RAG pipeline, highlighting the flow of patient information between different steps, such as retrieval, informativeness filtering, refinement, summarization, augmentation and final response generation.

### Clinician Assessment

To establish a human performance benchmark for diagnostic classification, six medical students were recruited: three interns and three residents, all with comparable levels of training and clinical experience. Each student was assigned a stratified random subset of cases (28 cases each, except for one student who received 29), with no overlap between subsets to prevent contamination. Stratification ensured that at least one rare and diagnostically challenging class, specifically C6 (diseases of immune dysregulation), C7 (autoinflammatory disorders), and C8 (phenocopies of IEI), was represented in every subset. This prevented any participant from receiving a disproportionately easier or harder set and ensured that each subset reflected the overall composition of the dataset. Cases were presented to participants via an online form, and participants were permitted to consult non-AI knowledge sources. All students had no prior exposure to the patients or their medical records.

### Evaluation Metrics

The reliability of each model was assessed by measuring the consistency of diagnostic classifications across three repeated generation runs, quantified using Fleiss’ kappa [46]. A score of 1 indicates perfect agreement across repeated runs. Model performance was evaluated using the weighted F1 score and the area under the receiver operating characteristic curve (AUC). Per-class ROC curves were computed using a one-vs-rest scheme, where each model’s output confidence score served as the decision score. A macro-averaged ROC curve was then obtained by averaging the per-class true positive rates (TPR) across a unified false positive rate (FPR) grid, and the macro-averaged AUC was computed accordingly. Where models were permitted to express diagnostic uncertainty, “I don’t know” responses were treated as incorrect classifications and counted as errors in the calculation of all validity metrics.

## Results

### Response Reliability

The consistency of class assignments generated by LLMs across three repeated runs and four different prompts strategies are summarized in Table 2. The models exhibited consistently high agreement across prompts, with the average Fleiss’s kappa values of 0.86 under minimal and 0.91 under full-form input. The variations for all models and input scenarios were minimum (*σ <0.05*). The Deepseek model achieved the lowest reliability and the Gemini achieved the best agreement in the full-form input (*κ = 0.98*). A notable observation is how model consistency increased with the full-form input compared to the minimal one. Looking at the average differences across prompts, particularly for Deepseek $\Delta =+0.15,\, (p<0.001)$, shows that the full-form performs more reliably.

**Table 2:** Fleiss’s kappa scores quantifies the agreement of LLM-generated class assignments across different input scenarios, prompting strategies and repeated runs. The values are averaged across three repeated runs

	Deepseek		Gemini		GPT-4o-Mini		LLama	
Record	Minimal	Full	Minimal	Full	Minimal	Full	Minimal	Full
Prompt 1	0.62	0.77	0.92	0.98	0.88	0.93	0.96	0.96
Prompt 2	0.64	0.78	0.92	0.98	0.93	0.94	0.94	0.96
Prompt 3	0.62	0.79	0.94	0.97	0.95	0.95	0.94	0.95
Prompt 4	0.67	0.79	0.88	0.98	0.99	0.95	0.95	0.96

It is also worth mentioning that, in Prompts 1 and 4, the model could respond with “I don’t know” however, only Deepseek produced responses indicating uncertainty: once in Prompt 1 and twice in Prompt 4, each occurring during a specific iteration for the same patient record.

### Baseline LLMs

**Fig. 3:**
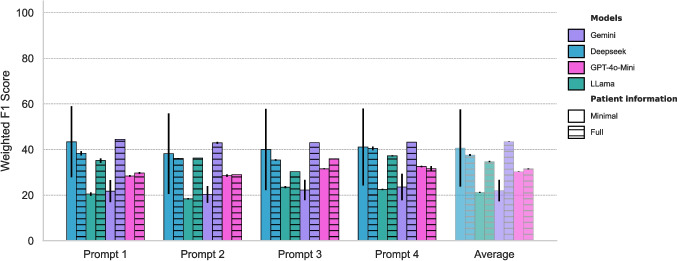
Classification performance of baseline LLMs. See Supplementary Table A.4 for more details

Figure 3 illustrates the weighted F1 scores used to evaluate the performance of baseline LLMs in IEI class-level categorization across four prompt strategies and two input scenarios (minimal and full-form). Weighted F1 scores are used for overall performance comparison, as macro-F1 is sensitive to the extremely small sample sizes in specific categories, such as C7 and C8 (*n=1*). Furthermore, as categories C9 and C10 contained no cases in this cohort, any model assignments to these classes were treated as errors and integrated into the aggregate metrics rather than per-class analyses. The Gemini model with full-form input, hereafter referred to as Gemini (full), achieved the highest mean weighted F1 ($43.39\, \%\, \pm \, 0.10$), followed by the Deepseek model under minimal-form input ($40.65\, \%\, \pm \, 0.56$), hereafter referred to as Deepseek (minimal).

In terms of the impact of the input scenario on validity, Gemini (full) demonstrated a major improvement, $\Delta =+21.43, \,CI (95\, \%) = 19.96 - 22.89,\, p<0.001$. LLama (full) also showed a statistically significant increase in weighted F1 score, $\Delta =+13.52, \,CI (95\, \%) = 8.86 - 18.17,\, p=0.011$. However, Deepseek and GPT-4o-Mini exhibited no significant differences between the input scenarios. Supplementary Tables A.4-A.6 present the classification performance of baseline LLMs per IEI classes using macro, and weighted F1 scores.

### RAG Strategies

**Fig. 4:**
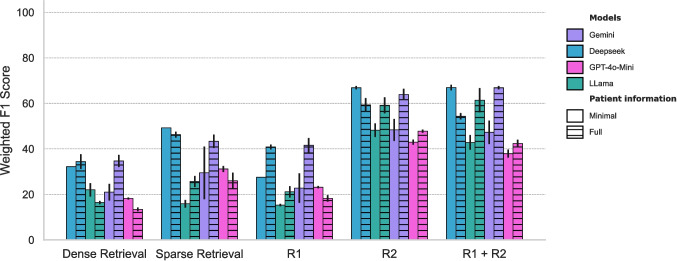
Performance comparison of the RAG strategies. See Supplementary Table B.7 for more details

Figure 4 presents the results obtained over three repeated runs using both minimal and full-form input. All results are limited to Prompt 4. The R1+R2 strategy, which integrates multi-step retrieval with informativeness and relevance filtering, achieved the highest overall score. The Deepseek (minimal) and the Gemini (full) models achieved the highest weighted F1 score of $66.94\, \%\, \pm 1.19$ and $66.90\, \%\, \pm 0.72$ respectively. Both models exhibited a positive performance compared to their baseline counterparts, $\Delta =+25.9, \,CI (95\, \%) = 6.8 - 45.0,\, p=0.116$ for Deepseek (minimal) and $\Delta =+23.7, \,CI (95\, \%) = 22.9 - 24.5,\, p<0.001$ for Gemini (full). Similarly, The LLama (full) model reached a higher weighted F1 score of $61.41\, \%\, \pm 5.30$ compared with its baseline of $37.19\, \%$ ($\Delta =+24.2,\, p=0.004$). Supplementary Tables B.7-B.9 present the classification performance of baseline LLMs for each IEI class in terms of macro and weighted F1 scores.

**Fig. 5:**
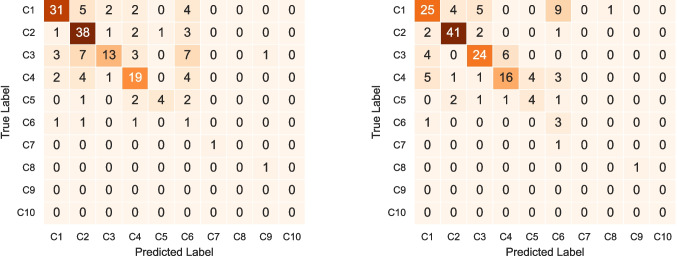
Confusion matrices comparing the classification performance of the Deepseek (minimal) (left) and Gemini (full) (right) in R1+R2 RAG strategy

**Fig. 6:**
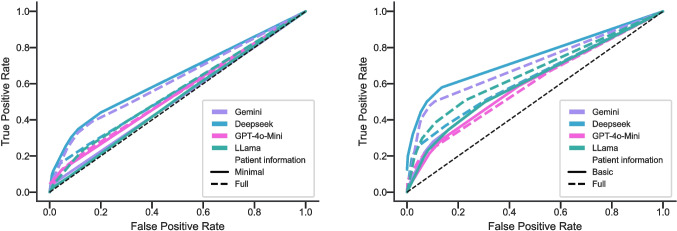
ROC curves of the baseline LLMs (left) and the RAG strategy R1+R2 (right)

To better compare the performance improvement of RAG models over baseline LLMs, the macro-averaged ROC curves for the R1+R2 strategy and baseline LLMs (Prompt 4) are shown in Fig. 6. The Deepseek (minimal) model achieved an AUC of $74\, \%$, while the Gemini (full) model followed with $70\, \%$. The complete AUC values are reported in Table 3. Figure 5 displays the confusion matrices for the Deepseek (minimal) and Gemini (full) models with R1+R2 RAG-enhanced strategy. Both models achieved higher classification performance in the C2 class compared to other IEI classes. Deepseek (minimal) showed the highest misclassification rate in C3, whereas Gemini (full) showed the highest misclassification rate in C1 (Fig. 6).

**Table 3:** AUC scores of the baseline LLMs and the best-performing RAG strategy

	Deepseek		Gemini		GPT-4o-Mini		LLama	
Record	Minimal	Full	Minimal	Full	Minimal	Full	Minimal	Full
LLM (Prompt 4)	0.64	0.56	0.51	0.62	0.54	0.54	0.51	0.56
RAG (R1+R2)	0.74	0.63	0.62	0.70	0.60	0.60	0.61	0.66

### Clinician-Based Evaluation

**Fig. 7:**
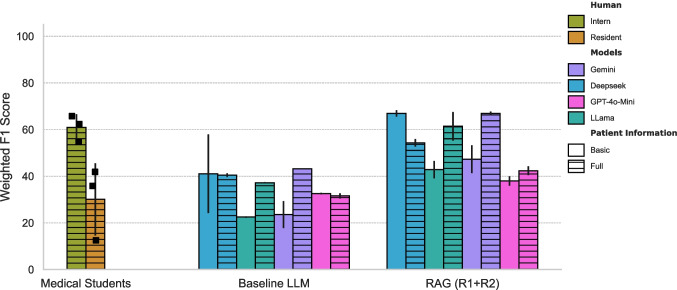
The classification performance of medical students, baseline LLMs, and RAG R1+R2 strategy in minimal and full-form inputs. Bars indicate mean weighted F1 score; error bars show standard deviation

Figure 7 presents a comparative analysis of classification performance across medical students, baseline LLMs, and retrieval-augmented models. The medical student evaluations, including two cohorts, interns and residents, were based on full-form input. The results for LLMs and the R1+R2 strategy are based on Prompt 4. As shown in Fig. 7, interns and residents achieved a mean weighted F1 score of $60.95 \pm 5.65\, \%$, and $30.06 \pm 15.51\, \%$, respectively. The highest intern weighted F1 was $65.76\, \%$, close to the Deepseek (minimal) and the Gemini (full) models.

## Discussion

Many primary care physicians may not be familiar with the details of each specific IEI class due to their rarity [19, 47]. Accessible resources may therefore help clinicians diagnose and classify IEIs more effectively [19]. The conventional process of evaluating a clinical evidence involves manual search and selection of relevant, high-quality evidence to identify and diagnose a case which has inherent limitations, such as inefficiency, and the possibility of missing important information. Given the increased usage of LLMs in serving as question answering systems in several medical domains, it seems necessary to evaluate the responses of these models in special scenarios, particularly due to the potential of hallucination, out-of-date information, as well as the lack of evidence-based and overconfident responses [48]. To address these limitations, this study evaluated the validity and reliability of the model responses in IEI classification. In addition, a quality and relevance filtering and refinement strategy has been proposed within the RAG pipeline to enhance model performance.

In clinical diagnostics, especially for rare diseases, model reliability is as critical as its accuracy. We found through the experiment that the response consistency was not significantly related to the prompt strategy and its difference between the models. Among the models, as previously reported in the literature [49], GPT-4o-Mini exhibited the highest prompt sensitivity in its minimal input scenario. On the other hand, additional input information led to higher scores for all models. The greatest effect was observed for the Deepseek model. In average, the highest consistency was found in Gemini with full-form input. This finding is consistent with Chimirri et al. study [50] showing diagnostic stability in LLMs for rare-disease cases. Recent studies have further highlighted the trade-off between validity and reliability [51, 52]. This suggests the need for prompt optimization strategies specific to each model, as different prompts affect models differently [53, 54].

The reluctance of LLMs to express uncertainty is a significant limitation. LLMs have an excessive tendency to be overconfident in their responses, even when they are wrong [55]. This kind of overconfidence bias, especially in medical applications, may pose risks because it causes the model to give even the wrong answers with high confidence [56, 57]. In our framework, we penalized “I don’t know” responses as incorrect to maintain a rigorous benchmark for diagnostic sensitivity. However, only Deepseek demonstrated even limited capability in expressing uncertainty. Diagnostic decisions for IEIs, require explicit acknowledgment of uncertainty and prevent model overconfidence due to its diagnostic complexity, particularly when incomplete information is inevitable. Studies show that expressing uncertainty can enhance user trust, satisfaction, and decision-making performance [58].

The comparison of baseline models shows that existing language models lack diagnostic knowledge in classifications of IEIs. Despite subtle differences in prompts, this finding contrasts with a prior study by Rider et al. [19]. Among our baseline models, the Gemini (full) model achieved the highest weighted F1 score using the full-form input, though it remained less than $50\, \%$, followed closely by the Deepseek (minimal) model. While the Deepseek model showed higher score variance, its AUC was the highest ($63.61\, \%$), marginally outperforming the Gemini model ($61.85\, \%$). Consistent with Yu et al. [59], our findings emphasize that baseline LLMs inherently lack domain-specific diagnostic reasoning, and that measurable improvement can be achieved through retrieval-augmented reasoning pipelines that integrate external medical knowledge which is a valuable approach for rare-disease diagnosis. A similar statement was raised in the Rider et al. [60] study, which emphasizes that the AI tools must be optimized and validated for different healthcare settings because their performance is affected by factors such as age, race, ethnicity, and available resources.

A reliable decision support system should be evidence-based, driven by a set of recognized guidelines, and designed to ensure access to right information [60]. In this study, the RAG strategy has been integrated in the proposed decision support pipeline to enhance the model’s capability and to get reliable and context-aware decisions. Using the hybrid method, which integrates semantic precision and content quality refinement strategies, led to significant improvements in the models. The Deepseek (minimal), Gemini (full), and LLama (full) models exceeded their baselines. These results are consistent with findings from other RAG-enhanced diagnostic studies, where retrieval substantially improved both validity and reliability [61–63]. In particular, the Deepseek model exhibited a $91.8\, \%$ reduction in standard deviation following RAG integration, reinforcing its potential for reliable IEI classification support. Similar consistency and diagnostic performance improvements have also been reported in recent graph-based RAG models for rare genetic disease diagnostics [64].

Beyond overall validity metrics, confusion matrix showed class-specific diagnostic trends. Class “Congenital defects of phagocyte number or function” showed the highest correct classification rate across all models, potentially reflecting more explicit clinical knowledge or stronger representation in the training or retrieval data. Conversely, persistent misclassification in classes “Combined immunodeficiencies with associated or syndromic features”, “Predominantly antibody deficiencies”, “Immunodeficiencies affecting cellular and humoral immunity”, “Defects in intrinsic and innate immunity”, and “Diseases of immune dysregulation” suggests challenges related to overlapping clinical features or diagnostic complexity. Most importantly, RAG substantially reduced misclassifications in classes “Combined immunodeficiencies with associated or syndromic features” and “Defects in intrinsic and innate immunity”, suggesting these categories particularly benefited from retrieval-based enhancement. For Class “Immunodeficiencies affecting cellular and humoral immunity”, the RAG strategy significantly improved classification performance, indicating that enhanced contextual knowledge likely resulted from targeted knowledge retrieval. The prevalence of “Combined immunodeficiencies with associated or syndromic features” in the dataset may have contributed to initial overfitting or biased classification, which RAG effectively countered.

Another notable observation was that the RAG-enhanced models outperformed the best intern student by a small margin. Residents, despite being more experienced and educated, achieved the lowest mean F1 score, which may reflect clinical pressures, knowledge fatigue, or limited exposure to rare IEI presentations during their training [65]. These disparities reflect broader trends in medical AI research, where human diagnostic performance can vary significantly due to contextual and systemic factors [66]. Also, this highlights a potential role for generative AI–based tools in decision support and medical education, particularly for diseases where training cases and educational resources are inherently limited. Therefore, LLMs, especially when augmented with external knowledge, can provide consistent, fatigue-free performance to complement clinical decision-making. This deployment can be associated with enhancements in procedural efficiency, user experience, and reduced workload [67]. Following this preliminary study, targeted educational interventions were implemented to improve the IEIs diagnostic skill of residents and interns. While observational evidence suggests these interventions were beneficial and led to higher guideline adherence, their direct impact has not been systematically studied.

These findings carry practical implications for IEI classification aids, health professional education, and future IEI research. The improved performance of RAG-enhanced models suggests that integrating LLMs with external medical knowledge bases could support clinical decision-making by facilitating early triage, narrowing the differential, and guiding targeted genetic testing, particularly in settings where access to immunology specialists is limited. While prospective validation in real-world clinical workflows remains necessary, these results position RAG-enhanced AI as a promising decision-support tool capable of improving the efficiency and accuracy of IEI class assignments. Additionally, the model-dependent nature of RAG suggests that healthcare organizations will need to carefully evaluate and optimize their chosen AI systems for their specific use cases and patient populations. The epidemiological variations observed across different populations further emphasize the importance of population-specific model validation and adaptation [68].

### Limitations

Despite these advantages, several limitations warrant acknowledgment. First, the dataset used may not represent the full spectrum of IEI complexity observed in clinical settings. This study relied on a single-center dataset of 169 records, and robust external validation across diverse, multi-center cohorts will be necessary to account for regional epidemiological variations and ensure the tool’s reliability across different patient populations. Furthermore, the absence of cases in classes C9 and C10 and the minimal representation of C7 and C8 limit our ability to reliably estimate per-class performance for these specific categories.

Second, comparisons between LLMs and medical students were conducted under non-identical conditions. While LLMs evaluated the full dataset, each student evaluated only a unique subset. Although stratified sampling was designed to ensure distributional equivalence across subsets, human and LLM performance were not assessed on an identical case set. Observed performance differences may therefore be partially influenced by case-level characteristics rather than reflecting purely skill-based distinctions. It should also be noted that this human evaluation serves as a preliminary benchmark to demonstrate that LLMs can perform at a comparable level to clinicians in low-resource settings, rather than as a definitive comparison. A fully controlled design, in which all participants evaluate the same set of cases, would be methodologically preferable and is recommended for future studies.

Third, the single-turn prompts employed do not reflect the iterative, multi-step reasoning processes typically used by specialists. Unlike clinicians, who dynamically request additional tests and refine their diagnostic reasoning through sequential information gathering, LLMs in this study received a fixed input without the ability to query for missing or complementary information. A more realistic evaluation framework would involve a step-by-step reasoning scenario, in which the LLM iteratively interacts with clinical data, more closely mimicking real diagnostic workflows. Such an agentic approach, where the LLM serves as the reasoning core of a decision support system, represents a promising direction for future IEI diagnostic tools.

Finally, the effectiveness of the retrieval process depends on retrieval quality, as irrelevant or incorrect context can mislead the models. Moreover, medical knowledge is not always available in a structured, machine-readable format [69], and its complexity and specialized terminology increase the risk of retrieving irrelevant or unhelpful documents. Several postprocessing steps are therefore required to prepare an extended knowledge base for use as a reliable retrieval source. Future work should prioritize the development of robust, high-quality medical knowledge bases tailored to rare disease contexts such as IEI.

## Conclusion

In this study, we demonstrate that retrieval-augmented large language models can provide consistent and improved class-level categorization of IEIs, supporting their potential role as a clinical decision-support tool. By incorporating external knowledge sources, retrieval-augmented generation overcomes key limitations of pretrained LLMs, particularly in rare and specialized medical domains where current, context-specific information is essential. However, the models’ tendency toward overconfidence necessitates careful implementation with appropriate human oversight, as well as further optimization and rigorous external validation. Our findings serve as a proof-of-concept for the potential of RAG-based tools to complement clinical decision-making, but they are not yet a substitute for specialist expertise. Future work will focus on incorporating uncertainty quantification and further optimizing prompt and retrieval strategies for IEI. We also plan to expand and refine knowledge sources and explore alternative knowledge representations, such as graph-based or rule-based structures, while ensuring that these technological advances translate into safe, equitable, and practical benefits for patients.

## Supplementary Information

Below is the link to the electronic supplementary material.

Supplementary file 1 (pdf 214 KB)

## Data Availability

No datasets were generated or analysed during the current study.

## References

[1] Notarangelo LD, Bacchetta R, Casanova J-L, Su HC. Human inborn errors of immunity: an expanding universe. Sci Immunol. 2020;5(49):eabb1662. 32651211 10.1126/sciimmunol.abb1662 PMC7647049

[2] Tangye SG, Al-Herz W, Bousfiha A, Cunningham-Rundles C, Franco JL, Holland SM, et al. Human inborn errors of immunity: 2022 update on the classification from the international union of immunological societies expert committee. J Clin Immunol. 2022;42(7):1473–507. 35748970 10.1007/s10875-022-01289-3 PMC9244088

[3] Poli MC, Aksentijevich I, Bousfiha AA, Cunningham-Rundles C, Hambleton S, Klein C, et al. Human inborn errors of immunity: 2024 update on the classification from the international union of immunological societies expert committee. J Human Immun. 2025;1(1):e20250003. 41608114 10.70962/jhi.20250003 PMC12829761

[4] Mohammadpour S, Emami H, Shokri S, Bagherzadeh R, Omari Shekaftik S, Hosseini Z. Primary immunodeficiency registry system: The minimum data set designing phase–a systematic review and quantitative delphi study. Health Sci Rep. 2025;8(7):e71015. 10.1002/hsr2.71015 PMC12241437 40642570

[5] Hu X, Ran AR, Nguyen TX, Szeto S, Yam JC, Chan CK, et al. What can gpt-4 do for diagnosing rare eye diseases? a pilot study. Ophthalmol Therapy. 2023;12(6):3395–402. 10.1007/s40123-023-00789-8 PMC10640532 37656399

[6] Weber MT, Noll R, Marchl A, Facchinello C, Grünewaldt A, Hügel C, et al. Medbot vs realdoc: efficacy of large language modeling in physician-patient communication for rare diseases. J Am Med Inform Assoc. 2025;32(5):775–83. 39998911 10.1093/jamia/ocaf034 PMC12012358

[7] Jeon S, Lee S-A, Chung H-S, Yun JY, Park EA, So M-K, et al. Evaluating the use of generative artificial intelligence to support genetic counseling for rare diseases. Diagnostics. 2025;15(6):672. 40150015 10.3390/diagnostics15060672 PMC11941130

[8] Rivière JG, Carot-Sans G, Piera-Jiménez J, De La Torre S, Cos X, Serra-Picamal X, et al. Development of an expert-based scoring system for early identification of patients with inborn errors of immunity in primary care settings-the pidcap project. J Clin Immunol. 2025;45(1):26. 10.1007/s10875-024-01825-3 PMC11493793 39432052

[9] Adams N, Hoehndorf R, Gkoutos GV, Hansen G, Hennig C. Pido: the primary immunodeficiency disease ontology. Bioinformatics. 2011;27(22):3193–9. 21949270 10.1093/bioinformatics/btr531

[10] Nikzad S, Johnson R, Scalchunes C, Rider NL. Quantifying the diagnostic odyssey burden among persons with inborn errors of immunity. J Clin Immunol. 2025;45(1):61. 39747782 10.1007/s10875-024-01855-x PMC11695393

[11] Barrera JAM, Guzmán SR, Cascajares EH, Garabedian EK, Fuleihan RL, Sullivan KE, et al. Who’s your data? primary immune deficiency differential diagnosis prediction via machine learning and data mining of the usidnet registry. Clin Immunol. 2023;255:109759. 37678719 10.1016/j.clim.2023.109759 PMC13498973

[12] Takao MMV, Carvalho LSF, Silva PGP, Pereira MM, Viana AC, da Silva MTN, et al. Artificial intelligence in allergy and immunology: comparing risk prediction models to help screen inborn errors of immunity. Int Arch Allergy Immunol. 2022;183(11):1226–30. 35973410 10.1159/000526204

[13] Ong M, Gilbert K, Aglan M, Barmettler S, Zhang A, Khairnar N, et al. Development of a computable phenotype for inborn errors of immunity: A machine learning approach, Annals of Allergy. Asthma Immunol. 2024;133(6):S64.

[14] Johnson B, Morales A, Facio F, Fresard L, McKnight D, Kobayashi Y, et al. The impact of machine learning models in reducing variants of uncertain significance for individuals from underrepresented populations who are undergoing testing for inborn errors of immunity. Clin Immunol. 2023;250:109469.

[15] Bellanti JA. Is it time for the a/i (allergist/immunologist) to embrace ai (artificial intelligence) in diagnosis and treatment of the inborn errors of immunity? In: Allergy and asthma proceedings. 2025. vol. 46. p. 354. 10.2500/aap.2025.46.250049 40958180

[16] Rivière JG, Palacín PS, Butte MJ. Proceedings from the inaugural artificial intelligence in primary immune deficiencies (aipid) conference. J Allergy Clin Immunol. 2024;153(3):637–42. 38224784 10.1016/j.jaci.2024.01.002 PMC11402388

[17] Rider N, Shin H, Roberts K. Text-mined data improves machine learning predictions for detecting inborn errors of immunity. Clin Immunol. 2023;250:109602.

[18] Roberts K, Chin AT, Loewy K, Pompeii L, Shin H, Rider NL. Natural language processing of clinical notes enables early inborn error of immunity risk ascertainment. J Allerg Clin Immunol Global. 2024;3(2):100224. 10.1016/j.jacig.2024.100224 PMC10910118 38439946

[19] Rider NL, Li Y, Chin AT, DiGiacomo DV, Dutmer C, Farmer JR, et al. Evaluating large language model performance to support the diagnosis and management of patients with primary immune disorders. J Allerg Clin Immunol. 2025. 10.1016/j.jaci.2025.02.004 PMC12229761 39956279

[20] Thirunavukarasu AJ, Ting DSJ, Elangovan K, Gutierrez L, Tan TF, Ting DSW. Large language models in medicine. Nat Med. 2023;29(8):1930–40. 37460753 10.1038/s41591-023-02448-8

[21] He K, Mao R, Lin Q, Ruan Y, Lan X, Feng M, et al. A survey of large language models for healthcare: from data, technology, and applications to accountability and ethics. Inf Fus. 2025;118:102963.

[22] Clusmann J, Kolbinger FR, Muti HS, Carrero ZI, Eckardt J-N, Laleh NG, et al. The future landscape of large language models in medicine. Commun Med. 2023;3(1):141. 37816837 10.1038/s43856-023-00370-1 PMC10564921

[23] Rouzrokh P, Khosravi B, Faghani S, Moassefi M, Shariatnia MM, Rouzrokh P, et al. A current review of generative ai in medicine: Core concepts, applications, and current limitations. Curr Rev Musculoskelet Med. 2025;1–21. 10.1007/s12178-025-09961-y PMC12185825 40304941

[24] Chen J, Lin H, Han X, Sun L. Benchmarking large language models in retrieval-augmented generation. In: Proceedings of the AAAI conference on artificial intelligence. 2024. vol. 38, p. 17754–17762.

[25] Xu X, Liu S, Zhu L, Long Y, Zeng Y, Lu X, et al. Development and evaluation of a retrieval-augmented large language model framework for enhancing endodontic education. Int J Med Informatics. 2025;106006. 10.1016/j.ijmedinf.2025.106006 40479778

[26] Mondillo G, Colosimo S, Perrotta A, Frattolillo V, Masino M, Martino M, et al. Artificial intelligence for solving pediatric clinical cases: A retrieval-augmented approach utilizing llama3. 2 and structured references. Int J Med Informatics. 2025;106027. 10.1016/j.ijmedinf.2025.106027 40561685

[27] Ke YH, Jin L, Elangovan K, Abdullah HR, Liu N, Sia ATH, et al. Retrieval augmented generation for 10 large language models and its generalizability in assessing medical fitness. NPJ Digit Med. 2025;8(1):187. 40185842 10.1038/s41746-025-01519-z PMC11971376

[28] Yan S-Q, Gu J-C, Zhu Y, Ling Z-H. Corrective retrieval augmented generation. 2024. arXiv preprint arXiv:2401.15884

[29] Salemi A, Zamani H. Evaluating retrieval quality in retrieval-augmented generation. In: Proceedings of the 47th international ACM SIGIR conference on research and development in information retrieval. 2024. p. 2395–2400.

[30] Fink A, Rau A, Reisert M, Bamberg F, Russe MF. Retrieval-augmented generation with large language models in radiology: From theory to practice. Radiol Artif Intell. 2025;7(4):e240790. 40464682 10.1148/ryai.240790

[31] Zarfati M, Soffer S, Nadkarni GN, Klang E. Retrieval-augmented generation: Advancing personalized care and research in oncology. Eur J Cancer. 2025;220:115341. 40068371 10.1016/j.ejca.2025.115341

[32] Gargari OK, Habibi G. Enhancing medical ai with retrieval-augmented generation: A mini narrative review. Digit Health. 2025;11:20552076251337176. 40343063 10.1177/20552076251337177 PMC12059965

[33] Liu S, McCoy AB, Wright A. Improving large language model applications in biomedicine with retrieval-augmented generation: a systematic review, meta-analysis, and clinical development guidelines. J Am Med Inform Assoc. 2025;32(4):605–15. 39812777 10.1093/jamia/ocaf008 PMC12005634

[34] Miao Y, Zhao Y, Luo Y, Wang H, Wu Y. Improving large language model applications in the medical and nursing domains with retrieval-augmented generation: Scoping review. J Med Internet Res. 2025;27:e80557. 41118646 10.2196/80557 PMC12587015

[35] Hugging Face, meta-llama/meta-llama-3-8b-instruct. https://huggingface.co/meta-llama/Meta-Llama-3-8B-Instruct. Accessed 05 Jul 2025.

[36] Guo D, Yang D, Zhang H, Song J, Zhang R, Xu R, Zhu Q, Ma S, Wang P, Bi X, et al. Deepseek-r1: Incentivizing reasoning capability in llms via reinforcement learning. 2025. arXiv preprint arXiv:2501.12948

[37] Menick J, Lu K, Zhao S, Wallace E, Ren H, Hu H, et al. Gpt-4o mini: advancing cost-efficient intelligence, Open AI: San Francisco. USA: CA; 2024.

[38] Team G, Georgiev P, Lei VI, Burnell R, Bai L, Gulati A, Tanzer G, Vincent D, Pan Z, Wang S, et al. Gemini 1.5: Unlocking multimodal understanding across millions of tokens of context. 2024. arXiv preprint arXiv:2403.05530

[39] Sullivan KE, Stiehm ER. Stiehm’s immune deficiencies: inborn errors of immunity. Academic Press. 2020.

[40] Robertson S, Zaragoza H, et al. The probabilistic relevance framework: Bm25 and beyond. Found Trends® Inf Retriev. 2009;3(4):333–389.

[41] Karpukhin V, Oguz B, Min S, Lewis PS, Wu L, Edunov S, Chen D, Yih W-t. Dense passage retrieval for open-domain question answering. In: EMNLP (1). 2020. p. 6769–6781.

[42] Balog K. Entity retrieval. Springer New York, New York, NY; 2018. p. 1326–31. 10.1007/978-1-4614-8265-9_80724

[43] Tran HD, Yates A. Dense retrieval with entity views. In: Proceedings of the 31st ACM international conference on information & knowledge management. 2022. p. 1955–64.

[44] Cohen R, Biswas R, de Melo G. Infact: Informativeness alignment for improved llm factuality. 2025. arXiv preprint arXiv:2505.20487

[45] Peng B, Galley M, He P, Cheng H, Xie Y, Hu Y, Huang Q, Liden L, Yu Z, Chen W, et al. Check your facts and try again: Improving large language models with external knowledge and automated feedback. 2023. arXiv preprint arXiv:2302.12813

[46] Gwet KL. Large-sample variance of fleiss generalized kappa. Educ Psychol Measur. 2021;81(4):781–90. 34267400 10.1177/0013164420973080 PMC8243202

[47] Orange JS, Seeborg FO, Boyle M, Scalchunes C, Hernandez-Trujillo V. Family physician perspectives on primary immunodeficiency diseases. Front Med. 2016;3:12. 10.3389/fmed.2016.00012 PMC4811961 27066486

[48] Shool S, Adimi S, Saboori Amleshi R, Bitaraf E, Golpira R, Tara M. A systematic review of large language model (llm) evaluations in clinical medicine. BMC Med Inform Decis Mak. 2025;25(1):117. 10.1186/s12911-025-02954-4 PMC11889796 40055694

[49] Balta KY, Javidan AP, Walser E, Arntfield R, Prager R. Evaluating the appropriateness, consistency, and readability of chatgpt in critical care recommendations. J Intensive Care Med. 2025;40(2):184–90. 39118320 10.1177/08850666241267871 PMC11639400

[50] Chimirri L, Caufield JH, Bridges Y, Matentzoglu N, Gargano M, Cazalla M, Chen S, Danis D, Dingemans AJ, Gehle K, et al. Consistent performance of large language models in rare disease diagnosis across ten languages and 4917 cases. EBioMedicine 2025;121. 10.1016/j.ebiom.2025.105957 PMC12552141 41092581

[51] Pugh SL, Chandler C, Cohen AS, Diaz-Asper C, Elvevåg B, Foltz PW. Assessing dimensions of thought disorder with large language models: The tradeoff of accuracy and consistency. Psychiatry Res. 2024;341:116119. 39226873 10.1016/j.psychres.2024.116119

[52] Davis J, Van Bulck L, Durieux BN, Lindvall C. The temperature feature of chatgpt: modifying creativity for clinical research. JMIR Hum Factors. 2024;11(1):e53559. 38457221 10.2196/53559 PMC10960206

[53] Wang L, Chen X, Deng X, Wen H, You M, Liu W, et al. Prompt engineering in consistency and reliability with the evidence-based guideline for llms. NPJ Digit Med. 2024;7(1):41. 38378899 10.1038/s41746-024-01029-4 PMC10879172

[54] Azimi I, Qi M, Wang L, Rahmani AM, Li Y. Evaluation of llms accuracy and consistency in the registered dietitian exam through prompt engineering and knowledge retrieval. Sci Rep. 2025;15(1):1506. 39789057 10.1038/s41598-024-85003-w PMC11718202

[55] Bentegeac R, Le Guellec B, Kuchcinski G, Amouyel P, Hamroun A. Token probabilities to mitigate large language models overconfidence in answering medical questions: Quantitative study. J Med Internet Res. 2025;27:e64348. 40882190 10.2196/64348 PMC12396779

[56] Abdar M, Pourpanah F, Hussain S, Rezazadegan D, Liu L, Ghavamzadeh M, et al. A review of uncertainty quantification in deep learning: Techniques, applications and challenges. Inf Fusion. 2021;76:243–97.

[57] Begoli E, Bhattacharya T, Kusnezov D. The need for uncertainty quantification in machine-assisted medical decision making. Nat Mach Intell. 2019;1(1):20–3.

[58] Xu Z, Song T, Lee Y-C. Confronting verbalized uncertainty: Understanding how llm’s verbalized uncertainty influences users in ai-assisted decision-making. Int J Hum Comput Stud. 2025;197:103455.

[59] Yu H, Chen T, Zhang X, Yang Y, Liu Q, Yang C, et al. Performance of large language models in diagnosing rare hematologic diseases and the impact of their diagnostic outputs on physicians: Combined retrospective and prospective study. J Med Internet Res. 2025;27:e77334. 41070713 10.2196/77334 PMC12511990

[60] Rider NL, Bastarache L, Anderson JT, Behrens EM, Chaimowitz N, Chandrakasan S, et al. Expert-based, institutional approaches for reducing the diagnostic odyssey of patients with ieis. J Allergy Clin Immunol: Practice. 2025. 10.1016/j.jaip.2025.03.040 40187490

[61] Kresevic S, Giuffrè M, Ajcevic M, Accardo A, Crocè LS, Shung DL. Optimization of hepatological clinical guidelines interpretation by large language models: a retrieval augmented generation-based framework. NPJ Digit Med. 2024;7(1):102. 38654102 10.1038/s41746-024-01091-y PMC11039454

[62] Cremaschi M, Ditolve D, Curcio C, Panzeri A, Spoto A, Maurino A. Decoding the mind: A rag-llm on icd-11 for decision support in psychology. Expert Syst Appl. 2025;279:127191.

[63] Amugongo LM, Mascheroni P, Brooks S, Doering S, Seidel J. Retrieval augmented generation for large language models in healthcare: A systematic review. PLOS Digital Health. 2025;4(6):e0000877. 40498738 10.1371/journal.pdig.0000877 PMC12157099

[64] Song J, Xu Z, He M, Feng J, Shen B. Graph retrieval augmented large language models for facial phenotype associated rare genetic disease. NPJ Digit Med. 2025;8(1):543. 40849403 10.1038/s41746-025-01955-x PMC12375027

[65] Goldman MP, Palladino LE, Malik RN, Powers EM, Rudd AV, Aronson PL, et al. A workplace procedure training cart to augment pediatric resident procedural learning. Pediatr Emerg Care. 2022;38(2):e816–20. 35100781 10.1097/PEC.0000000000002397

[66] Shan G, Chen X, Wang C, Liu L, Gu Y, Jiang H, et al. Comparing diagnostic accuracy of clinical professionals and large language models: Systematic review and meta-analysis. JMIR Med Inform. 2025;13(1):e64963. 40279517 10.2196/64963 PMC12047852

[67] Artsi Y, Sorin V, Glicksberg BS, Korfiatis P, Nadkarni GN, Klang E. Large language models in real-world clinical workflows: A systematic review of applications and implementation. Front Digit Health. 2025;7:1659134. 41098649 10.3389/fdgth.2025.1659134 PMC12519456

[68] Abolhassani H, Kiaee F, Tavakol M, Chavoshzadeh Z, Mahdaviani SA, Momen T, et al. Fourth update on the iranian national registry of primary immunodeficiencies: integration of molecular diagnosis. J Clin Immunol. 2018;38(7):816–32. 30302726 10.1007/s10875-018-0556-1

[69] Liu S, McCoy AB, Chen Q, Wright A. Integrating rule-based nlp and large language models for statin information extraction from clinical notes. Int J Med Informatics. 2025;106104. 10.1016/j.ijmedinf.2025.106104 PMC12705237 40925145

