# Supplementary material for: Retrieval-Augmented Language Models for Clinical Decision Support in the Classification of Inborn Errors of Immunity
Source: J Clin Immunol. 2026 Jun 5;46(1):79. doi: 10.1007/s10875-026-02035-9 (PMC13461685; doi:10.1007/s10875-026-02035-9)
Supplement: Supplementary file 1 [file 10875_2026_2035_MOESM1_ESM.pdf]

Appendix A. Classification performance of baseline LLMs

Table A.1: Classification performance of baseline LLMs (Weighted F1, %). Results are reported as mean ± standard deviation.

	Deepseek		Gemini		GPT-4o-Mini		LLama	
**Record**	Minimal	Full	Minimal	Full	Minimal	Full	Minimal	Full
Prompt 1	43.37 ± 15.57	38.30 ± 0.90	21.75 ± 4.82	44.43 ± 0.00	28.41 ± 0.38	29.71 ± 0.39	20.45 ± 0.72	35.15 ± 0.93
Prompt 2	38.15 ± 17.64	36.07 ± 0.18	20.31 ± 3.66	42.95 ± 0.41	28.55 ± 0.52	28.99 ± 0.00	18.39 ± 0.31	36.29 ± 0.00
Prompt 3	40.01 ± 17.82	35.39 ± 0.36	22.24 ± 4.42	43.00 ± 0.00	31.55 ± 0.24	35.89 ± 0.00	23.48 ± 0.50	30.25 ± 0.00
Prompt 4	41.07 ± 16.84	40.44 ± 0.80	23.56 ± 5.78	43.19 ± 0.00	32.56 ± 0.35	31.58 ± 1.12	22.49 ± 0.32	37.19 ± 0.25
Average	40.65 ± 16.93	37.55 ± 0.40	21.97 ± 4.66	43.39 ± 0.10	30.27 ± 0.08	31.54 ± 0.19	21.20 ± 0.21	34.72 ± 0.27

Table A.2: Classification performance of baseline LLMs (Macro F1, %). Results are reported as mean ± standard deviation.

	Deepseek		Gemma		GPT-4o-Mini		LLaMA	
**Record**	Minimal	Full	Minimal	Full	Minimal	Full	Minimal	Full
Prompt 1	20.55 ± 6.54	17.97 ± 0.40	9.83 ± 2.23	22.26 ± 0.00	13.39 ± 0.35	15.93 ± 0.16	10.42 ± 0.39	19.85 ± 0.43
Prompt 2	16.78 ± 7.85	16.09 ± 0.08	10.29 ± 1.88	23.11 ± 0.19	13.59 ± 0.15	14.85 ± 0.00	9.49 ± 0.19	19.84 ± 0.00
Prompt 3	24.18 ± 12.36	14.35 ± 0.14	9.92 ± 1.90	21.90 ± 0.00	13.99 ± 0.14	16.96 ± 0.00	12.43 ± 0.25	19.19 ± 0.00
Prompt 4	21.33 ± 9.29	18.86 ± 0.34	10.52 ± 2.67	22.59 ± 0.00	14.56 ± 0.13	14.27 ± 0.42	11.84 ± 0.19	23.27 ± 0.12
Average	20.71 ± 8.91	16.82 ± 0.17	10.14 ± 2.17	22.46 ± 0.05	13.89 ± 0.06	15.50 ± 0.07	11.04 ± 0.13	20.54 ± 0.13

Table A.3: Classification performance of baseline LLMs (Per Class F1, %). Results are reported as mean ± standard deviation.

		Deepsee		Gemmini		GPT-4o-Mini		LLaMA	
**Prompt**	**Class**	**Minimal**	**Full**	**Minimal**	**Full**	**Minimal**	**Full**	**Minimal**	**Full**
Prompt 1	1	51.59 ± 10.28	41.21 ± 0.52	32.64 ± 1.89	35.02 ± 0.55	36.68 ± 1.75	43.10 ± 1.60	20.00 ± 0.00	23.61 ± 1.20
Prompt 1	2	58.37 ± 18.93	49.14 ± 5.25	79.31 ± 1.31	45.55 ± 1.34	28.65 ± 13.11	21.96 ± 0.46	81.19 ± 0.00	66.92 ± 2.28
Prompt 1	3	29.85 ± 17.62	16.67 ± 2.89	19.84 ± 0.28	32.79 ± 0.00	12.28 ± 6.08	0.00 ± 0.00	64.52 ± 0.00	31.58 ± 0.00
Prompt 1	4	38.43 ± 22.91	0.00 ± 0.00	22.86 ± 0.00	0.00 ± 0.00	10.88 ± 2.36	18.33 ± 1.03	11.76 ± 0.00	20.00 ± 0.00
Prompt 1	5	22.91 ± 9.32	17.86 ± 6.19	0.00 ± 0.00	30.00 ± 0.00	0.00 ± 0.00	0.00 ± 0.00	33.33 ± 0.00	16.67 ± 0.00
Prompt 1	6	0.00 ± 0.00	0.00 ± 0.00	7.06 ± 0.14	0.00 ± 0.00	0.00 ± 0.00	0.00 ± 0.00	11.76 ± 0.00	0.00 ± 0.00
Prompt 1	7	0.00 ± 0.00	0.00 ± 0.00	0.00 ± 0.00	0.00 ± 0.00	0.00 ± 0.00	0.00 ± 0.00	0.00 ± 0.00	0.00 ± 0.00
Prompt 1	8	0.00 ± 0.00	0.00 ± 0.00	0.00 ± 0.00	0.00 ± 0.00	0.00 ± 0.00	0.00 ± 0.00	0.00 ± 0.00	0.00 ± 0.00
Prompt 2	1	48.82 ± 11.17	42.10 ± 0.10	35.99 ± 0.87	35.22 ± 0.00	36.05 ± 1.63	41.11 ± 0.30	19.58 ± 1.09	7.02 ± 0.00
Prompt 2	2	52.15 ± 24.50	50.94 ± 2.13	86.06 ± 0.60	46.67 ± 0.00	26.50 ± 10.86	15.90 ± 0.18	82.00 ± 0.00	68.63 ± 0.00
Prompt 2	3	22.44 ± 21.27	14.63 ± 0.00	15.38 ± 0.00	29.51 ± 0.00	8.77 ± 3.04	0.00 ± 0.00	64.87 ± 0.61	43.81 ± 0.00
Prompt 2	4	36.23 ± 21.61	0.00 ± 0.00	0.00 ± 0.00	0.00 ± 0.00	10.97 ± 2.43	18.92 ± 1.03	6.25 ± 0.00	39.29 ± 0.00
Prompt 2	5	5.56 ± 9.62	14.65 ± 0.63	0.00 ± 0.00	22.22 ± 0.00	0.00 ± 0.00	0.00 ± 0.00	18.18 ± 0.00	0.00 ± 0.00
Prompt 2	6	0.00 ± 0.00	0.00 ± 0.00	7.41 ± 0.00	0.00 ± 0.00	0.00 ± 0.00	0.00 ± 0.00	17.14 ± 0.00	0.00 ± 0.00
Prompt 2	7	0.00 ± 0.00	0.00 ± 0.00	0.00 ± 0.00	0.00 ± 0.00	0.00 ± 0.00	0.00 ± 0.00	0.00 ± 0.00	0.00 ± 0.00
Prompt 2	8	0.00 ± 0.00	0.00 ± 0.00	0.00 ± 0.00	0.00 ± 0.00	0.00 ± 0.00	0.00 ± 0.00	0.00 ± 0.00	0.00 ± 0.00
Prompt 3	1	47.22 ± 9.56	41.95 ± 0.31	23.24 ± 0.83	33.57 ± 0.00	39.48 ± 1.22	41.71 ± 0.38	18.39 ± 0.00	20.37 ± 0.00
Prompt 3	2	56.38 ± 23.75	60.08 ± 0.59	87.55 ± 0.54	62.69 ± 0.00	31.64 ± 15.66	23.30 ± 1.87	78.00 ± 0.00	60.47 ± 0.00
Prompt 3	3	24.47 ± 22.30	15.26 ± 0.22	26.67 ± 0.00	41.79 ± 0.00	5.56 ± 0.00	7.14 ± 0.00	59.70 ± 0.00	23.38 ± 0.00
Prompt 3	4	33.77 ± 25.41	0.00 ± 0.00	0.00 ± 0.00	0.00 ± 0.00	12.59 ± 2.66	27.28 ± 0.54	16.22 ± 0.00	9.30 ± 0.00
Prompt 3	5	21.08 ± 8.62	22.66 ± 0.75	0.00 ± 0.00	31.58 ± 0.00	0.00 ± 0.00	0.00 ± 0.00	33.33 ± 0.00	40.00 ± 0.00
Prompt 3	6	0.00 ± 0.00	0.00 ± 0.00	6.06 ± 0.00	0.00 ± 0.00	0.00 ± 0.00	0.00 ± 0.00	13.33 ± 0.00	0.00 ± 0.00
Prompt 3	7	55.56 ± 50.92	0.00 ± 0.00	0.00 ± 0.00	0.00 ± 0.00	0.00 ± 0.00	0.00 ± 0.00	0.00 ± 0.00	0.00 ± 0.00
Prompt 3	8	0.00 ± 0.00	0.00 ± 0.00	0.00 ± 0.00	0.00 ± 0.00	0.00 ± 0.00	0.00 ± 0.00	0.00 ± 0.00	0.00 ± 0.00
Prompt 4	1	49.61 ± 10.73	42.25 ± 0.15	30.31 ± 1.36	33.63 ± 0.66	40.67 ± 0.59	39.10 ± 0.59	22.47 ± 0.00	12.83 ± 0.12
Prompt 4	2	57.08 ± 20.91	59.81 ± 1.13	85.48 ± 1.93	52.11 ± 3.22	33.56 ± 14.69	24.85 ± 0.25	80.41 ± 0.00	67.11 ± 0.77
Prompt 4	3	25.05 ± 17.57	20.00 ± 0.00	26.87 ± 0.35	37.90 ± 0.34	8.77 ± 3.04	3.87 ± 0.04	63.49 ± 0.00	37.13 ± 0.21
Prompt 4	4	35.35 ± 26.50	0.00 ± 0.00	21.05 ± 0.00	0.00 ± 0.00	11.67 ± 5.77	26.86 ± 1.11	0.00 ± 0.00	35.71 ± 0.00
Prompt 4	5	20.00 ± 5.77	23.53 ± 0.00	0.00 ± 0.00	19.05 ± 0.00	0.00 ± 0.00	0.00 ± 0.00	42.86 ± 0.00	33.33 ± 0.00
Prompt 4	6	5.93 ± 5.14	0.00 ± 0.00	6.06 ± 0.00	0.00 ± 0.00	0.00 ± 0.00	0.00 ± 0.00	16.67 ± 0.00	0.00 ± 0.00
Prompt 4	7	16.67 ± 28.87	0.00 ± 0.00	0.00 ± 0.00	0.00 ± 0.00	0.00 ± 0.00	0.00 ± 0.00	0.00 ± 0.00	0.00 ± 0.00
Prompt 4	8	0.00 ± 0.00	0.00 ± 0.00	0.00 ± 0.00	0.00 ± 0.00	0.00 ± 0.00	0.00 ± 0.00	0.00 ± 0.00	0.00 ± 0.00

Appendix B. Classification performance of RAG strategies

Table B.4: Performance comparison of the RAG strategies (Weighted F1, %). Results are reported as mean ± standard deviation.

	Deepseek		Gemini		GPT-4o-Mini		LLama	
**Record**	Minimal	Full	Minimal	Full	Minimal	Full	Minimal	Full
Dense Retrieval	32.18 ± 0.00	34.42 ± 3.20	20.99 ± 3.63	34.70 ± 2.65	18.22 ± 0.46	13.43 ± 0.81	22.00 ± 2.94	16.38 ± 0.70
Sparse Retrieval	49.25 ± 0.00	46.26 ± 1.26	29.48 ± 11.56	43.31 ± 2.98	31.15 ± 1.29	26.02 ± 3.49	15.87 ± 1.66	25.67 ± 2.41
R1	27.49 ± 0.00	40.83 ± 1.06	22.77 ± 6.47	41.55 ± 3.21	23.16 ± 0.53	18.26 ± 1.44	15.31 ± 0.53	21.15 ± 2.48
R2	66.91 ± 0.75	59.35 ± 2.97	48.34 ± 4.82	63.90 ± 2.51	42.95 ± 1.13	47.78 ± 0.66	48.20 ± 2.97	59.15 ± 3.51
R1+R2	66.94 ± 1.19	54.31 ± 1.38	47.29 ± 5.17	66.90 ± 0.72	37.94 ± 1.74	42.35 ± 1.65	42.83 ± 3.23	61.41 ± 5.30

Table B.5: Performance comparison of the RAG strategies (Macro F1, %). Results are reported as mean ± standard deviation.

	Deepseek		Gemini		GPT-4o-Mini		LLama	
**Record**	Minimal	Full	Minimal	Full	Minimal	Full	Minimal	Full
Dense Retrieval	23.97 ± 0.00	20.82 ± 1.42	12.37 ± 4.58	22.45 ± 4.35	16.06 ± 0.93	12.28 ± 1.49	7.51 ± 1.14	15.34 ± 1.72
Sparse Retrieval	17.16 ± 0.00	17.11 ± 1.85	14.02 ± 3.77	18.35 ± 2.86	9.98 ± 0.29	7.61 ± 0.32	13.76 ± 1.67	10.68 ± 0.78
R1	14.10 ± 0.00	21.65 ± 1.21	12.80 ± 4.90	23.56 ± 3.30	13.04 ± 0.23	11.50 ± 0.78	9.59 ± 0.43	13.19 ± 1.42
R2	38.07 ± 0.37	35.27 ± 3.00	32.64 ± 3.75	33.95 ± 1.89	24.14 ± 2.16	24.36 ± 2.24	31.95 ± 2.57	36.34 ± 3.71
R1+R2	48.79 ± 0.75	30.43 ± 2.26	21.82 ± 2.03	37.45 ± 1.30	19.12 ± 1.59	24.68 ± 1.12	26.37 ± 2.14	38.24 ± 2.17

Table B.6: Classification performance of RAG strategies (Per Class F1, %). Results are reported as mean ± standard deviation.

		Deepsee		Gemmini		GPT-4o-Mini		LLaMA	
**Prompt**	**Class**	**Minimal**	**Full**	**Minimal**	**Full**	**Minimal**	**Full**	**Minimal**	**Full**
Dense Retrieval	1	50.67 ± 0.00	43.34 ± 2.24	43.07 ± 3.09	25.51 ± 5.91	44.91 ± 1.95	41.74 ± 1.64	40.73 ± 1.48	38.96 ± 3.58
Dense Retrieval	2	69.77 ± 0.00	78.95 ± 2.03	40.59 ± 28.99	72.58 ± 3.92	54.84 ± 2.48	35.41 ± 8.33	12.66 ± 5.46	26.29 ± 6.74
Dense Retrieval	3	36.73 ± 0.00	29.91 ± 4.68	18.79 ± 14.93	54.93 ± 4.99	14.47 ± 2.63	27.38 ± 9.39	2.50 ± 2.89	27.84 ± 3.28
Dense Retrieval	4	45.07 ± 0.00	41.62 ± 1.90	17.27 ± 7.57	25.43 ± 2.62	0.00 ± 0.00	0.00 ± 0.00	7.41 ± 2.74	12.98 ± 2.85
Dense Retrieval	5	26.67 ± 0.00	0.00 ± 0.00	6.90 ± 7.98	19.55 ± 0.91	30.33 ± 4.87	0.00 ± 0.00	0.00 ± 0.00	2.38 ± 4.76
Dense Retrieval	6	10.81 ± 0.00	3.45 ± 3.98	0.00 ± 0.00	6.86 ± 0.42	0.00 ± 0.00	0.00 ± 0.00	0.00 ± 0.00	14.26 ± 6.23
Dense Retrieval	7	0.00 ± 0.00	0.00 ± 0.00	0.00 ± 0.00	0.00 ± 0.00	0.00 ± 0.00	0.00 ± 0.00	0.00 ± 0.00	0.00 ± 0.00
Dense Retrieval	8	0.00 ± 0.00	0.00 ± 0.00	0.00 ± 0.00	25.00 ± 50.00	0.00 ± 0.00	0.00 ± 0.00	0.00 ± 0.00	0.00 ± 0.00
Sparse Retrieval	1	37.78 ± 0.00	34.89 ± 1.93	37.50 ± 3.07	35.43 ± 1.82	42.53 ± 0.43	37.99 ± 0.48	43.36 ± 1.45	41.14 ± 2.77
Sparse Retrieval	2	55.56 ± 0.00	65.23 ± 9.16	23.72 ± 13.75	51.23 ± 6.07	23.91 ± 1.67	5.23 ± 3.98	20.78 ± 6.44	8.23 ± 3.16
Sparse Retrieval	3	5.26 ± 0.00	8.22 ± 2.99	15.42 ± 4.30	39.70 ± 6.16	2.86 ± 3.30	5.23 ± 0.13	15.49 ± 6.49	8.13 ± 2.89
Sparse Retrieval	4	32.43 ± 0.00	27.99 ± 9.36	4.50 ± 3.32	7.00 ± 6.74	0.00 ± 0.00	0.00 ± 0.00	6.87 ± 5.24	1.47 ± 2.94
Sparse Retrieval	5	0.00 ± 0.00	15.00 ± 10.00	10.00 ± 11.55	43.17 ± 4.08	0.00 ± 0.00	20.00 ± 0.00	12.35 ± 10.05	28.82 ± 3.18
Sparse Retrieval	6	15.38 ± 0.00	6.88 ± 0.58	11.69 ± 14.15	0.00 ± 0.00	0.00 ± 0.00	0.00 ± 0.00	0.00 ± 0.00	0.00 ± 0.00
Sparse Retrieval	7	8.00 ± 0.00	0.00 ± 0.00	9.30 ± 2.34	0.00 ± 0.00	10.53 ± 0.00	0.00 ± 0.00	11.20 ± 1.14	0.00 ± 0.00
Sparse Retrieval	8	0.00 ± 0.00	0.00 ± 0.00	0.00 ± 0.00	0.00 ± 0.00	0.00 ± 0.00	0.00 ± 0.00	0.00 ± 0.00	0.00 ± 0.00
R1	1	37.97 ± 0.00	39.41 ± 1.30	44.71 ± 3.31	40.38 ± 7.84	51.91 ± 1.31	42.78 ± 0.49	44.58 ± 1.63	39.03 ± 3.84
R1	2	47.83 ± 0.00	71.41 ± 1.49	28.65 ± 13.39	58.47 ± 6.56	31.31 ± 1.23	12.99 ± 3.50	7.32 ± 2.75	19.46 ± 3.64
R1	3	5.56 ± 0.00	39.46 ± 3.54	12.08 ± 11.08	44.33 ± 4.12	0.00 ± 0.00	11.20 ± 4.46	0.00 ± 0.00	13.06 ± 6.33
R1	4	12.70 ± 0.00	10.54 ± 2.76	0.00 ± 0.00	26.75 ± 7.44	0.00 ± 0.00	0.00 ± 0.00	3.14 ± 3.89	10.14 ± 9.31
R1	5	22.86 ± 0.00	22.04 ± 0.79	16.93 ± 19.91	22.34 ± 1.88	21.06 ± 0.49	25.00 ± 0.00	21.72 ± 1.87	23.81 ± 1.68
R1	6	0.00 ± 0.00	6.11 ± 4.95	0.00 ± 0.00	11.37 ± 2.50	0.00 ± 0.00	0.00 ± 0.00	0.00 ± 0.00	0.00 ± 0.00
R1	7	0.00 ± 0.00	0.00 ± 0.00	0.00 ± 0.00	0.00 ± 0.00	0.00 ± 0.00	0.00 ± 0.00	0.00 ± 0.00	0.00 ± 0.00
R1	8	0.00 ± 0.00	0.00 ± 0.00	0.00 ± 0.00	0.00 ± 0.00	0.00 ± 0.00	0.00 ± 0.00	0.00 ± 0.00	0.00 ± 0.00
R2	1	74.12 ± 2.23	51.25 ± 1.53	62.63 ± 8.95	52.02 ± 5.76	54.45 ± 1.07	54.71 ± 1.47	63.65 ± 3.85	64.79 ± 0.77
R2	2	84.49 ± 1.86	89.38 ± 0.50	65.53 ± 11.68	86.39 ± 2.21	64.62 ± 1.85	69.90 ± 2.33	53.79 ± 4.76	74.44 ± 1.92
R2	3	52.26 ± 3.63	58.77 ± 8.14	38.92 ± 8.90	74.82 ± 2.26	38.02 ± 5.30	60.37 ± 1.70	34.16 ± 10.83	54.56 ± 3.46
R2	4	60.31 ± 1.70	39.50 ± 9.82	21.98 ± 6.13	47.27 ± 9.07	0.00 ± 0.00	0.00 ± 0.00	40.02 ± 6.44	43.95 ± 19.15
R2	5	57.14 ± 0.00	48.08 ± 2.22	34.52 ± 39.91	51.67 ± 6.14	64.27 ± 1.61	44.51 ± 2.02	55.65 ± 12.95	60.86 ± 4.62
R2	6	2.38 ± 4.76	11.64 ± 2.85	11.81 ± 13.68	27.36 ± 5.43	0.00 ± 0.00	0.00 ± 0.00	0.00 ± 0.00	0.00 ± 0.00
R2	7	50.00 ± 0.00	0.00 ± 0.00	58.33 ± 9.62	0.00 ± 0.00	20.00 ± 23.09	0.00 ± 0.00	8.33 ± 16.67	0.00 ± 0.00
R2	8	0.00 ± 0.00	0.00 ± 0.00	0.00 ± 0.00	0.00 ± 0.00	0.00 ± 0.00	0.00 ± 0.00	0.00 ± 0.00	0.00 ± 0.00
R1+R2	1	75.01 ± 2.84	50.99 ± 2.16	67.91 ± 11.39	64.62 ± 3.17	58.83 ± 2.87	52.67 ± 0.68	65.15 ± 1.88	65.90 ± 2.51
R1+R2	2	76.05 ± 1.39	89.36 ± 2.54	70.14 ± 6.13	87.77 ± 1.30	60.26 ± 2.03	63.30 ± 5.16	54.96 ± 5.44	77.77 ± 8.04
R1+R2	3	55.60 ± 3.11	51.78 ± 3.67	24.59 ± 6.86	73.66 ± 1.64	21.66 ± 4.03	47.78 ± 3.57	6.38 ± 6.26	59.41 ± 11.73
R1+R2	4	65.28 ± 0.58	24.53 ± 10.19	21.00 ± 3.64	50.24 ± 7.78	0.00 ± 0.00	0.00 ± 0.00	41.29 ± 8.45	45.54 ± 14.87
R1+R2	5	59.34 ± 2.54	32.66 ± 3.31	34.58 ± 5.65	34.26 ± 8.53	34.94 ± 2.77	33.66 ± 3.68	43.20 ± 4.37	57.30 ± 13.81
R1+R2	6	7.81 ± 0.69	8.21 ± 2.90	0.00 ± 0.00	26.46 ± 7.03	0.00 ± 0.00	0.00 ± 0.00	0.00 ± 0.00	0.00 ± 0.00
R1+R2	7	100.00 ± 0.00	0.00 ± 0.00	0.00 ± 0.00	0.00 ± 0.00	0.00 ± 0.00	0.00 ± 0.00	0.00 ± 0.00	0.00 ± 0.00
R1+R2	8	0.00 ± 0.00	0.00 ± 0.00	0.00 ± 0.00	0.00 ± 0.00	0.00 ± 0.00	0.00 ± 0.00	0.00 ± 0.00	0.00 ± 0.00

Appendix C. Example prompt used in the Study

Prompt Structure

Role

You are an expert immunologist tasked with diagnosing a patient.

Guidance

Evaluate the following patient information for potential primary immunodeficiency. Determine the most likely primary immunodeficiency diagnosis (*Inborn Errors of Immunity*).

Start with the age of onset, then consider:

- location and types of infections,
- associated symptoms (autoimmune or syndromic features),
- and other relevant information.

If Diagnosis is Unclear

If you don't know the answer, explicitly state that you do not know.

Input

`{Patient information}`

Classification List

- Immunodeficiencies affecting cellular and humoral immunity
- Combined immunodeficiencies with associated or syndromic features
- Predominantly antibody deficiencies
- Diseases of immune dysregulation
- Congenital defects of phagocyte number or function
- Defects in intrinsic and innate immunity
- Autoinflammatory disorders
- Complement deficiencies
- Bone Marrow Failure
- Phenocopies of inborn errors of immunity

Appendix D. Categorization thresholds and ranges

Table D.7: Categorization thresholds for hematological parameters

Parameter	Condition / Threshold	Category
**Lymphocytes**	Age < 365 days and $Lym_{count} < 3000$	Lymphopenia
	Age ≥ 365 days and $Lym_{count} < 1000$	Lymphopenia
	Otherwise	Normal
**Neutrophils**	$PMN_{count} < 500$	Severe Neutropenia
	$500 \leq PMN_{count} < 1000$	Moderate Neutropenia
	$1000 \leq PMN_{count} < 1600$	Mild Neutropenia
	$1600 \leq PMN_{count} \leq 10000$	Normal
	$PMN_{count} > 10000$	Neutrophilia
**NBT (%)**	$NBT > 90$	Normal
	$10 \leq NBT \leq 90$	Suspicious
	$NBT < 10$	Abnormal
**Eosinophils**	$EO_{count} < 500$	Eosinophilia
	$EO_{count} \geq 500$	Normal
**Platelets**	$Plt < 150$	Low
	$Plt \geq 150$	Normal

Table D.8: Normal ranges of immunoglobulins (IgG, IgM, IgA) by age

Age	IgG (mg/dL)	IgM (mg/dL)	IgA (mg/dL)
Newborn	831–1231	6–16	0–3
1-3 month	311–549	19–41	8–34
4-6 month	241–613	26–60	10–46
7-12 month	442–880	31–77	19–55
13-24 month	553–971	35–81	26–74
25-36 month	709–1075	42–80	34–108
3-5 years	701–1157	38–74	66–120
6-8 years	667–1179	40–90	79–169
9-11 years	889–1359	46–112	71–191
12-16 years	822–1070	39–79	85–211
Adults	853–1463	72–126	139–261

Table D.9: IgE level categorization thresholds

Category	IgE Level (IU/mL)
Normal	$IgE < 500$
Mildly Increased	$500 \leq IgE < 1000$
High	$1000 \leq IgE < 2000$
Very High	$IgE \geq 2000$

Table D.10: Normal ranges for lymphocyte subsets (CD markers) by age

Age (days)	CD19 (%)	CD3 (%)	CD16/56 (%)	CD4 (%)	CD8 (%)
0–90	6–32	53–84	4–18	35–64	12–28
91–180	11–41	51–77	3–14	35–56	12–23
181–360	14–37	49–76	3–15	31–56	12–24
361–720	16–35	53–75	3–15	32–51	14–30
721–2160	14–33	56–75	4–17	28–47	16–30
2161–4320	13–27	60–76	4–17	31–47	18–35
4321–6480	6–23	56–84	3–22	31–52	18–35

Appendix E. Raw JSON structure for entity-based dense retrieval (R1)

Listing 1: Hierarchy of clinical related entity groups

```json
{
  "Family History": [
    "Family history of immunodeficiency",
    "Number of affected family members",
    "Family history of early deaths",
    "Other family history details"
  ],
  "Malignancies": [
    "Cancer types",
    "Other malignancies"
  ],
  "Inflammatory and Autoimmune Diseases": [
    "Inflammatory diseases",
    "Autoimmune diseases",
    "Other inflammatory/autoimmune diseases"
  ],
  "Patient Appearance and Symptoms": [
    "Growth and development issues",
    "Mucosal and dental findings",
    "Other appearance-related findings"
  ],
  "Patient's Autoimmune/Related Conditions": [
    "Specific conditions",
    "Other autoimmune-related conditions"
  ],
  "Allergies": [
    "Food allergies",
    "Drug allergies",
    "Environmental allergies",
    "Other allergies"
  ],
  "Organ-Specific Problems": [
    "Respiratory system",
    "Eye conditions",
    "Urinary system anomalies",
    "Cardiac issues",
    "Hematological issues",
    "Gastrointestinal issues",
    "Rheumatological issues",
    "Musculoskeletal system",
    "Neurological issues",
    "Dermatological issues",
    "Endocrine system",
    "Other organ-specific problems"
  ],
  "Physical Examination Findings": [
    "Height",
    "Weight",
    "Head Circumference",
    "Cardiovascular Examination",
    "Respiratory Examination",
    "Skin Examination",
    "Developmental Status",
    "Developmental Interventions"
  ],
```

```
  "Disability Information": [
    "Disability Status",
    "Age at Disability Onset",
    "Type of Disabling Condition"
  ],
  "Other Complications": [
    "Other complications"
  ]
}
```

Listing 2: Hierarchy of demographic related entity groups

```
{
    "Basic Information": [
      "Gender",
      "Ethnicity",
      "Nationality",
      "Age",
      "Place of Residence (Urban/Rural)"
    ],
    "Family and Birth Information": [
      "Number of Siblings",
      "Birth Order",
      "Type of Mother's Pregnancy (e.g., Single, Twin, IVF)",
      "Type of Delivery (e.g., Normal Vaginal Delivery, C-section)",
      "Birth Weight",
      "Birth Height",
      "Head Circumference at Birth",
      "Time to Umbilical Cord Separation",
      "Consanguinity (Relationship Between Parents)"
    ],
    "Family Health Information": [
      "Number of Affected Family Members",
      "Age of Death",
      "Cause of Death"
    ],
    "Disease Timeline": [
      "Age at Symptom Onset",
      "Age at Diagnosis"
    ]
}
```

Listing 3: Hierarchy of genetic related entity groups

```
{
    "Genetic Testing": [
      "Gene",
      "OMIM",
      "Mutation",
      "Type of Mutation",
      "Zygosity",
      "Inheritance",
      "Method",
      "Affected Domain",
      "Prediction Severity",
      "Medical Severity",
      "Consanguinity",
      "Genetic Diagnosis",
      "Genetic Defect"
    ]
  }
```

Listing 4: Hierarchy of immune related entity groups

```
{
    "Vaccine Reaction Characteristics": [
      "Vaccine Type",
      "Time of Reaction"
    ],
    "Vaccine Type": [
      "Diphtheria",
      "Tetanus",
      "HIB Polysaccharide",
      "Protein Conjugated HIB",
      "Isohemagglutinin-Anti A",
      "Isohemagglutinin-Anti B",
      "Chicken Pox",
      "Measles",
      "Mumps",
      "Rubella",
      "PHI X 174 or Other Neoantigen",
      "Anti-HIV Test",
      "PPT-Type III allergen test",
      "DHR Test"
    ],
    "Other Vaccination/Immunization Information": []
  }
```

Listing 5: Hierarchy of infection related entity groups

```
{
    "Infection Details": [
        "Infection Name",
        "Location of Infection",
        "Type of Infection",
        "Microorganism Identified",
        "Onset Timing",
    ],
    "Microbiological Characteristics": [
        "Gram Staining",
        "Antibiotic Susceptibility",
        "Resistance Genes",
        "Viral Subtype",
        "Fungal Species",
    ],
    "Etiology and Risk Factors": [
        "Cause of Infection"
    ],
    "Clinical Course of Infection": [
        "Severity of Infection",
        "Duration of Infection",
        "Hospitalization Requirement due to Infection",
        "Recurrence Count of Infection",
        "Complications from Infection",
        "Mortality or Morbidity Outcome of Infection",
        "Response to Infection Treatment",
        "Additional Infection-Related Clinical Details"
    ],
    "Treatment Information": [
        "Antibiotics Used",
        "Other Treatments",
        "Treatment Outcome"
```

```
    ],
    "Diagnostic Workup": [
        "Laboratory Test Results",
        "Radiology Findings",
        "Pathology Findings",
        "Genetic Testing Results",
        "Diagnostic Procedures Performed"
}
```

Listing 6: Hierarchy of laboratory tests related entity groups

```
{
  "Complete Blood Count (CBC)": [
    "WBC",
    "Platelets",
    "RBC",
    "Hemoglobin",
    "Lymphocytes",
    "PMN - polymorphonuclear leukocyte",
    "Eosinophils",
    "Basophils",
    "Neutrophil count",
    "Monocytes"
  ],
  "Cellular Immunology": [
    "Absolute Lymphocyte Count",
    "CD3 T Cells",
    "CD4 Helper T Cells",
    "CD8 Cytotoxic T Cells",
    "CD19 B Cells",
    "CD20 B Cells",
    "CD56/CD16 NK Cells"
  ],
  "CD4+ T Cell Subsets": [
    "Naive",
    "Total Memory",
    "Central Memory",
    "Effector Memory"
  ],
  "CD8+ T Cell Subsets": [
    "Naive",
    "Total Memory",
    "Central Memory",
    "Effector Memory",
    "Senescent"
  ],
  "Memory B Cells": [
    "CD27+ B Cells (Total Memory B)",
    "CD27+IgM+ B Cells (IgM Memory B)",
    "CD27+IgD- B Cells (Switched Memory B)"
  ],
  "Humoral Immunology": [
    "IgG",
    "IgA",
    "IgM",
    "IgE",
    "IgD",
    "IgG1",
    "IgG2",
    "IgG3",
    "IgG4"
  ],
  "Genetic Test": [],
  "Lymphocyte Function Tests": [
    "PHA (Phytohemagglutinin)",
    "ConA (Concanavalin A) Test",
    "PWM (Pokeweed Mitogen) Test"
  ],
  "Antigen Responses": [
```

```
    "Anti-CD3 Stimulation",
    "Tetanus",
    "Diphtheria",
    "Candida",
    "Alloantigen (MLC - Mixed Lymphocyte Culture)",
    "NK Cell Cytotoxicity Test"
  ],
  "Skin Testing": [
    "Tetanus",
    "Candida"
  ],
  "Complement Function Tests": [
    "CH50",
    "AH50"
  ],
  "Autoantibodies": [
    "Anti-neutrophil Antibody",
    "Anti-platelet Antibody",
    "Direct Coombs Positive",
    "Other Autoantibodies"
  ],
  "other laboratory tests":[]
}
```
